# Using the Morris Water Maze to Assess Spatial Learning and Memory in Weanling Mice

**DOI:** 10.1371/journal.pone.0124521

**Published:** 2015-04-17

**Authors:** Christopher D. Barnhart, Dongren Yang, Pamela J. Lein

**Affiliations:** Department of Molecular Biosciences, School of Veterinary Medicine, University of California Davis, Davis, CA, United States of America; Université Pierre et Marie Curie, FRANCE

## Abstract

Mouse models have been indispensable for elucidating normal and pathological processes that influence learning and memory. A widely used method for assessing these cognitive processes in mice is the Morris water maze, a classic test for examining spatial learning and memory. However, Morris water maze studies with mice have principally been performed using adult animals, which preclude studies of critical neurodevelopmental periods when the cellular and molecular substrates of learning and memory are formed. While weanling rats have been successfully trained in the Morris water maze, there have been few attempts to test weanling mice in this behavioral paradigm even though mice offer significant experimental advantages because of the availability of many genetically modified strains. Here, we present experimental evidence that weanling mice can be trained in the Morris water maze beginning on postnatal day 24. Maze-trained weanling mice exhibit significant improvements in spatial learning over the training period and results of the probe trial indicate the development of spatial memory. There were no sex differences in the animals’ performance in these tasks. In addition, molecular biomarkers of synaptic plasticity are upregulated in maze-trained mice at the transcript level. These findings demonstrate that the Morris water maze can be used to assess spatial learning and memory in weanling mice, providing a potentially powerful experimental approach for examining the influence of genes, environmental factors and their interactions on the development of learning and memory.

## Introduction

Neurodevelopmental disabilities, including autism spectrum disorders (ASD), attention-deficit hyperactivity disorder (ADHD), schizophrenia, learning disabilities, intellectual disability (also known as mental retardation), and sensory impairments, affect 10–15% of all births in the United States [1,2], and the prevalence of at least some of these disorders is increasing worldwide [3]. Given the tremendous costs exacted on the affected individual, their families, and society [4–6], there is an urgent need to identify and characterize factors that confer risk for these neurodevelopmental disabilities. Preclinical models, and in particular mouse models, have and will continue to play an important role in elucidating both genetic and environmental factors that influence normal and pathological processes of relevance to neurodevelopmental disabilities [7–10]. Experimental advantages offered by mouse models include not only the availability of a large number of genetically modified mouse strains but also the ability to study complex behaviors with face validity to the clinical phenotypes associated with neurodevelopmental disabilities [11], including learning and memory [12–14].

A widely used model for studying learning and memory behavior in mice is the Morris water maze (MWM), which specifically assesses spatial learning and memory [11,15]. This task has the advantage of being acquired quickly without pre-training or restriction of food and water. Moreover, learning, memory and factors that influence these behaviors, such as visual acuity, motor function and motivation, can be dissociated by manipulating the testing protocol [16]. However, the majority of studies of mice in the MWM have used adult animals. Because of the significant qualitative and quantitative differences between the developing and mature nervous systems [17], studies in adult animals may not reveal mechanisms by which genetic and/or environmental factors alter cognitive development [18]. For example, studies of juvenile rats exposed developmentally to polychlorinated biphenyls (PCBs) indicated deficits in learning and memory in PCB-exposed animals that correlated with decreased activity of choline acetyltransferase (ChAT) in the hippocampus and forebrain [19]. In contrast, studies of adult rats exposed developmentally to PCBs exhibited cognitive deficits in the absence of any effects on ChAT activity [20], reinforcing the idea that adults are not necessarily good models for identifying processes that influence cognitive development in young animals [18].

In rats, water maze learning develops immediately after weaning [21], and the MWM has been successfully employed to study the effects of alcohol [22], lead [23], chlorpyrifos [24], and PCBs [25] on cognitive function in weanling rats. A recent study demonstrated that mice exhibit spatial learning in the MWM when training begins at postnatal day 35 (PND 35) [26], suggesting that mice may also develop water maze learning shortly after weaning. Thus, the goal of our study was to determine whether the MWM could be adapted to assess spatial learning and memory in weanling mice. Moreover, since MWM training is associated with increased dendritic arborization and synaptogenesis [25,27], we also measured transcript levels of genes associated with synaptic plasticity. Our data demonstrate that weanling mice exhibit spatial learning and memory in the MWM and that MWM training increases at least a subset of molecular biomarkers of synaptic plasticity.

## Materials and Methods

### Animals

This study was carried out in strict accordance with the recommendations in the Guide for the Care and Use of Laboratory Animals of the National Institutes of Health. The protocol was approved by the Institutional Animal Care and Use Committee of the University of California, Davis (protocol number 15410). All animals were treated with regard for alleviation of suffering. Timed-pregnant C57BL/6 dams were purchased from Charles River Laboratories (Hollister, CA) and housed individually in standard plastic shoebox cages in a temperature-controlled (20 ± 1°C) room on a normal 12-h light-dark cycle. Food (LabDiet, St. Louis, MO) and autoclaved water were provided *ad libitum*. Litters were redistributed at postnatal day 2 (PND 2) to 8 pups per litter with roughly equal numbers of males and females. Pups were weaned at PND 21 and behavioral testing began at PND 24, with at most 1 male and 1 female pup selected from each litter for behavioral testing (e.g., the litter was the statistical unit of measure for behavioral studies).

### Morris water maze (MWM)

MWM testing was conducted in a round white pool 94 cm in diameter and 31 cm deep. The pool was filled to a depth of 30 cm with water made opaque with white non-toxic water-based tempura paint. Pool temperature was maintained at 25 ± 0.5°C by addition of warm water. The escape platform was a 25-cm^2^ Plexiglas square, placed in the center of one quadrant of the pool, 15 cm from the pool’s edge and submerged 1 cm beneath the water surface. The platform remained in the same position throughout the learning trials and visual cue tests and was removed from the pool during the probe test. Several distal extra-maze cues (a traffic cone, a colorful poster, and two black-and-white construction paper designs) were placed around the pool and these remained in the same position throughout the training and testing periods.

A trial began by placing the mouse on the platform for 20 s to allow orientation to extra-maze cues. After orientation, mice were gently lowered tail-first into the pool facing the wall at one of three positions, each at the center of the wall of a different quadrant not housing the platform. After the mouse was released, the researcher retreated away from the pool to a constant position within the room, serving as an additional distal visual cue. The SMART digital tracking system (Version 2.5, Panlab, Barcelona, Spain) simultaneously began recording the trial. Maximum swim time was set to 60 s. If the mouse located the platform before 60 s had passed, it was immediately removed from the pool. If the platform was not located after 60 s of swimming, the mouse was gently guided to the platform and allowed to re-orient to the distal visual cues for an additional 20 s before being removed from the pool. After removal from the pool, mice were manually dried with a terrycloth towel and placed in a warming cage (consisting of a heating pad set to low underneath a typical shoebox cage) for at least 5 min before returning to the home cage. Mice were visually inspected to ensure thorough dryness. Mice were tested in two trials per day with an inter-trial interval of approximately 30 min. All testing was conducted at roughly the same time each day in order to minimize variability in performance due to time of day.

To examine spatial reference memory, a probe test was administered 24 h after the last training session. During the probe test, the platform was removed from the pool and the mouse was allowed to swim freely for 1 min. A visual cue test was conducted 30 min after the probe test to assess sensorimotor ability and motivation [16]. For this test, the platform was set 1 cm above the water level and marked with black tape so that the mice could locate the platform using a local visual stimulus rather than relying on spatial orientation to extra-maze cues.

After completion of the visual cue test, all tracks from all trials were analyzed for a number of behavioral parameters using SMART software (Panlab). The resultant behavioral data were statistically analyzed as described below.

### RNA isolation and reverse transcription

Twenty-four h after completion of the visual cue test, MWM-trained weanlings were euthanized by cervical dislocation and brains immediately harvested. A day later, brains were harvested from non-maze-trained littermate controls that were treated identically with the exception of not being trained in the MWM. Brains were dissected on ice using sterile tools to obtain the cortices, cerebella, and hippocampi, which were snap frozen on dry ice. Brain tissues were homogenized by passing through a sterile 18G 1-1/2 inch needle 10 times, and total RNA was isolated from these homogenates using the RNEasy kit (Qiagen, Venlo, Netherlands). Genomic DNA was digested by incubation with recombinant DNAse I (Invitrogen, Carlsbad, CA). Total RNA (1 μg of each sample) was reverse transcribed to cDNA using the SuperScript III first strand synthesis system (Invitrogen). The concentration and purity of the resultant cDNA were determined using a Nanodrop spectrophotometer (NanoDrop, Wilmington, DE). cDNA concentrations were between 800–1100 µg/µL and the 260/280 and 260/230 ratios for the cDNA were all above 1.9.

### Quantitative PCR (qPCR)

Primer and probe sets specific for the target genes spinophilin (Spn), activity-regulated cytoskeleton-associated protein (ARC), neurogranin (RC3), Homer1a, Homer1b/c were designed using PrimerBlast (NCBI, Bethesda, MD) and PrimerQuest software (IDT, Coralville, IA). Specificity of the primers and probes were confirmed by nucleotide BLAST (NCBI, Bethesda, MD). The primer and probe sequences are provided in Table 1. The 18S rRNA genomic control was purchased from Eurogentec (Eurogentec, Seraing, Belgium). For each sample, 800 ng of cDNA was amplified using Taqman Universal PCR Master Mix (Life Sciences, Grand Island, NY) and a 7500 Fast Real-Time PCR System (Applied Biosystems, Foster City, CA). Dilution curves were run in each plate to determine amplification efficiencies. The concentration of forward and reverse primers (300 nM) and probe (100 nM) were determined in pilot studies. Thermal cycles consisted of an initial incubation with uracil-N-glycosylase (UNG) to remove uracil-containing PCR products (2 minutes at 50°C). This was followed by UNG deactivation, activation of the DNA polymerase, and an initial denaturation of sample cDNA (10 min at 95°C). qPCR cycling conditions were as follows: 40 cycles of denaturation (30 s at 95°C), annealing (1 min at 50–55°C) and extension (30 s at 72°C).

**Table 1:** Primer and probe sequences for synaptic plasticitygenes.

Gene (Full name)	Primer/Probe	Sequence
ARC (Activity-regulated cytoskeleton-associated protein)	Forward Primer	5’-ACGATCTGGCTTCCTCATTCTGCT-3’
	Reverse Primer	5’-AGGTTCCCTCAGCATCTCTGCTTT-3’
	Probe	5’-/56-FAM/AGTGTCCAGGGCTCTTTGGGTAATCA/36-TAMRASp /-3’
RC3 (Neurogranin)	Forward Primer	5’- GCCAGACGACGATATTCTTGACATC-3’
	Reverse Primer	5’-TTTATCTTCTTCCTCGCCATGTG-3’
	Probe	5’-/56-FAM/CCCGGAGCCAACGCCGCT/36-TAMRASp /-3’
SPN (Spinophilin)	Forward Primer	5’-AAGGCGGCCCACCATAA-3’
	Reverse Primer	5’-GCCCATCTGCAGGAACATACTT-3’
	Probe	5’-/56-FAM/TATGGCTCCAACGTCCA/36-TAMRASp/-3’
Homer1a	Forward Primer	5’-GCATTGCCATTTCCACATAGG-3’
	Reverse Primer	5’-ATGAACTTCCATATTTATCCACCTTACTT-3’
	Probe	5’-/56-FAM/ACACATTCAATTCAGCAATCATGA/36-TAMRASp /-3’
Homer1b/c	Forward Primer	5’-ACACCCGATGTGACACAGAACT-3’
	Reverse Primer	5’-TCAGCCTCCCAGTGTTTGCT-3’
	Probe	5’-/56-FAM/CGAACCCAGGCCCTCTCTCATGCT/3BHQ1 /-3’

C_t_ values were determined using the 7500 Fast System SDS software (Applied Biosystems, Foster City, CA), and were normalized to 18S rRNA within the same sample. To determine the fold-change in expression of target genes of interest, relative transcript expression between control and trained animals was calculated using the Pfaffl equation [28]:

R=(ETarget)ΔCt,Target(control−trained)(ERef)ΔCt,Ref(control−trained) (1)

where R refers to the fold-change in expression of the target gene in trained *versus* untrained control samples normalized to the reference gene. E_target_ refers to efficiency of target gene amplification and E_ref_ refers to the efficiency of amplification of the reference gene. The amplification efficiency for each gene was calculated for each brain region from the cDNA dilution curve for that particular gene using the formula $E=10^{-\left[1/\;slope\right]}$, where slope refers to the slope of the line generated by plotting C_t_ values against log (cDNA concentration). Efficiencies were calculated from dilution curves with an R^2^ of at least 0.985 that spanned at least 3 orders of magnitude. Amplification efficiency values ranged between 94–105%, with the exception of Homer1b/c in cortical samples (108.2%) and ARC in cortical samples (108.7%). C_t_ refers to the amplification cycle in which the reporter fluorescence exceeds a manually defined threshold. The ΔC_t_ value for each gene was determined by subtracting the average C_t_ value of each target gene in a specific brain region of the control animals from the average C_t_ value of the same gene in samples from the same brain region of the MWM-trained animals. Additional computational and statistical analysis was performed using REST2009 software (Qiagen) as previously described [29], which performs a randomized analysis of raw C_t_ data to provide statistical comparisons of gene expression between trained animals and non-maze-trained control littermates [28].

### Statistical analysis

Data analyzed by repeated measures ANOVA were first assessed for normality and sphericity using Shapiro-Wilks and Mauchly’s tests, respectively. These tests confirmed that repeated measures data were normally distributed, but some data violated the assumption of sphericity, in which case the Greenhouse-Geisser correction was used to correct the F statistic and assess significance. Data analyzed by ANOVA and t tests were assessed for normality and homogeneity of variance with Shapiro-Wilks test and Levene’s test, respectively. All data analyzed by ANOVA and t tests were normally distributed, but not all data had equal variances. Data with equal variances were assessed *post hoc* using the least significant difference (LSD) test, while data that violated Levene’s test were assessed using Welch’s test to ensure significance of the ANOVA and Games-Howell was used *post hoc* to identify differences between groups. Behavioral data from the training period were analyzed using repeated measures ANOVA. Data from the probe test were analyzed using one-way ANOVA. Data from the visual cue test were analyzed using a two-tailed unpaired t-test or repeated measures ANOVA. All behavioral data were analyzed in SPSS (version 22, IBM, Armonk, NY). Effect sizes and power calculations are included in the Results section and figure legends. Fold-changes in gene expression between MWM-trained and non-maze-trained weanlings were calculated from C_t_ values from qPCR experiments using the Pfaffl equation and analyzed for statistical significance using REST2009 software (Qiagen) as previously described [29].

## Results

### Weanling mice exhibit spatial learning and memory with MWM training

In the MWM task, the animal is required to find a hidden platform to escape from swimming in a pool of water. To accomplish this task, the animal forms a “spatial orientation map” in the brain using visual stimuli from extra-maze cues in the testing room. During training, learning is assessed by the amount of time elapsed before the animal climbs onto the platform to escape the water (escape latency) and by the percentage of time or path length spent in the quadrant housing the platform (target quadrant). These data were determined to be normally distributed. Analysis by repeated measures two-way ANOVA indicated no sex differences in any of the behavioral parameters reported below over the course of training, therefore, data from males and females were combined in all of the analyses discussed below.

**Fig 1:**
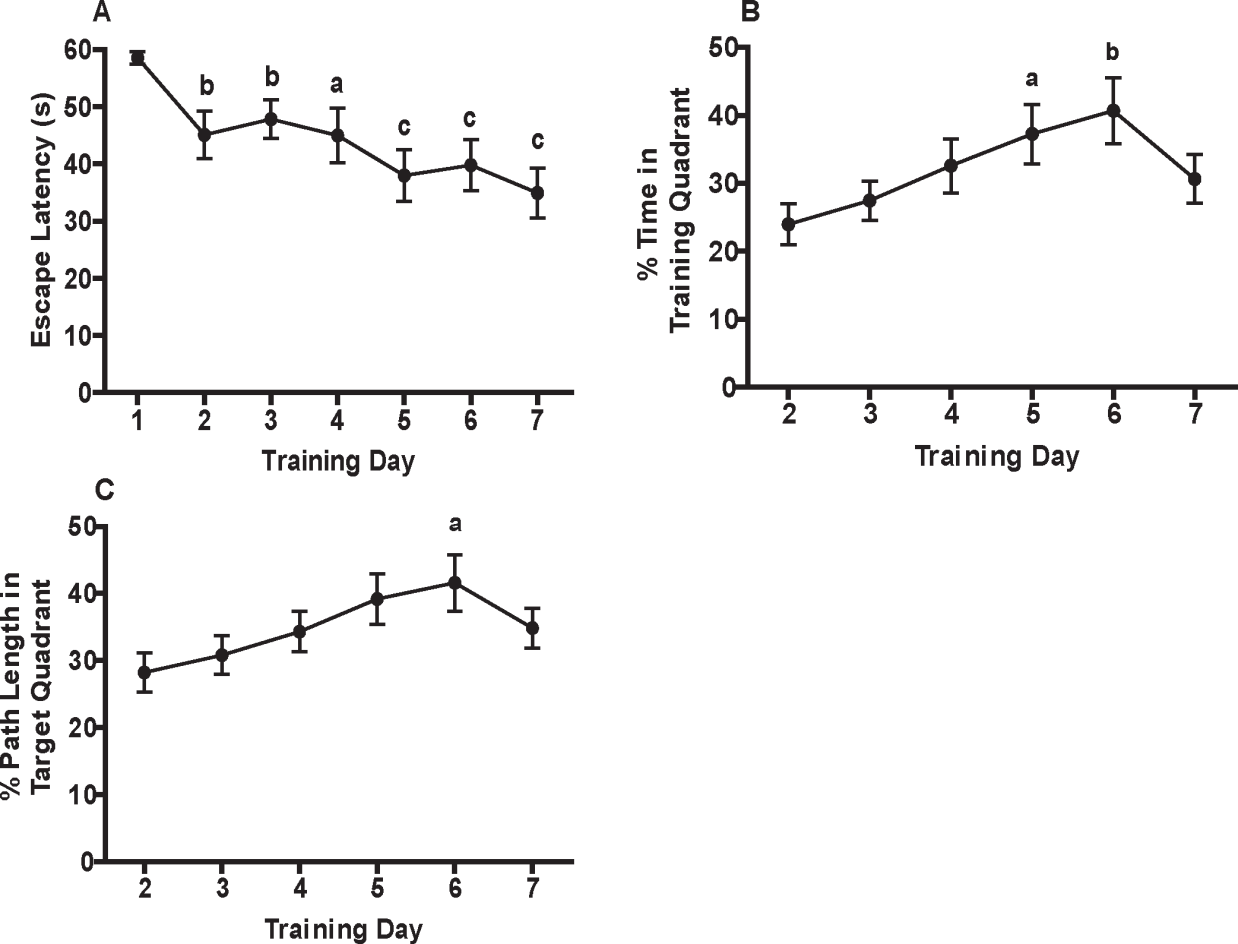
Weanling mice exhibit spatial learning in the Morris water maze (MWM). Spatial learning was assessed as a function of training day with respect to the following parameters: (A) escape latency, (B) percentage of time spent in the target quadrant, and (C) percentage of total path length spent in the target quadrant. Data are presented as the mean ± SEM (n = 16 animals). Since sex differences were not identified for any of the behavioral parameters shown in this Fig., data from males and females were combined to calculate mean values. Significantly different from training d 1 (A) or d 2 (B and C) at ^a^ *p* < 0.05, ^b^ *p* < 0.01, ^c^ *p* < 0.001 as determined using repeated measures ANOVA with LSD *post hoc* test. Effect sizes: partial η^2^ for latency = 0.31, partial η^2^ for % time = 0.21; partial η^2^ for % path length = 0.17. Power: 99% for latency, 94% for % time, 84% for % path length. Note that escape latency was the only data collected on the first day of training because of a computer malfunction in collecting data on the first training day.

Escape latency decreased over the 7 d training period (Fig 1A). Repeated-measures one-way ANOVA identified a significant reduction in escape latency with training (*F*(6,90) = 6.58, *p* < 0.0001). Weanlings also spent a significantly increased percentage of swimming time (*F*(5,75) = 4.08, *p* = 0.003) and path length (*F*(5,75) = 3.03, *p* = 0.02) in the target quadrant over the course of training (Fig 1B and 1C). The effect sizes for these parameters ranged between medium and large (for latency, partial η^2^ = 0.31; for % time, partial η^2^ = 0.21; for % path length, partial η^2^ = 0.17). The power calculated by SPSS was 99% for latency, 94% for % time, and 84% for % path length.

**Fig 2:**
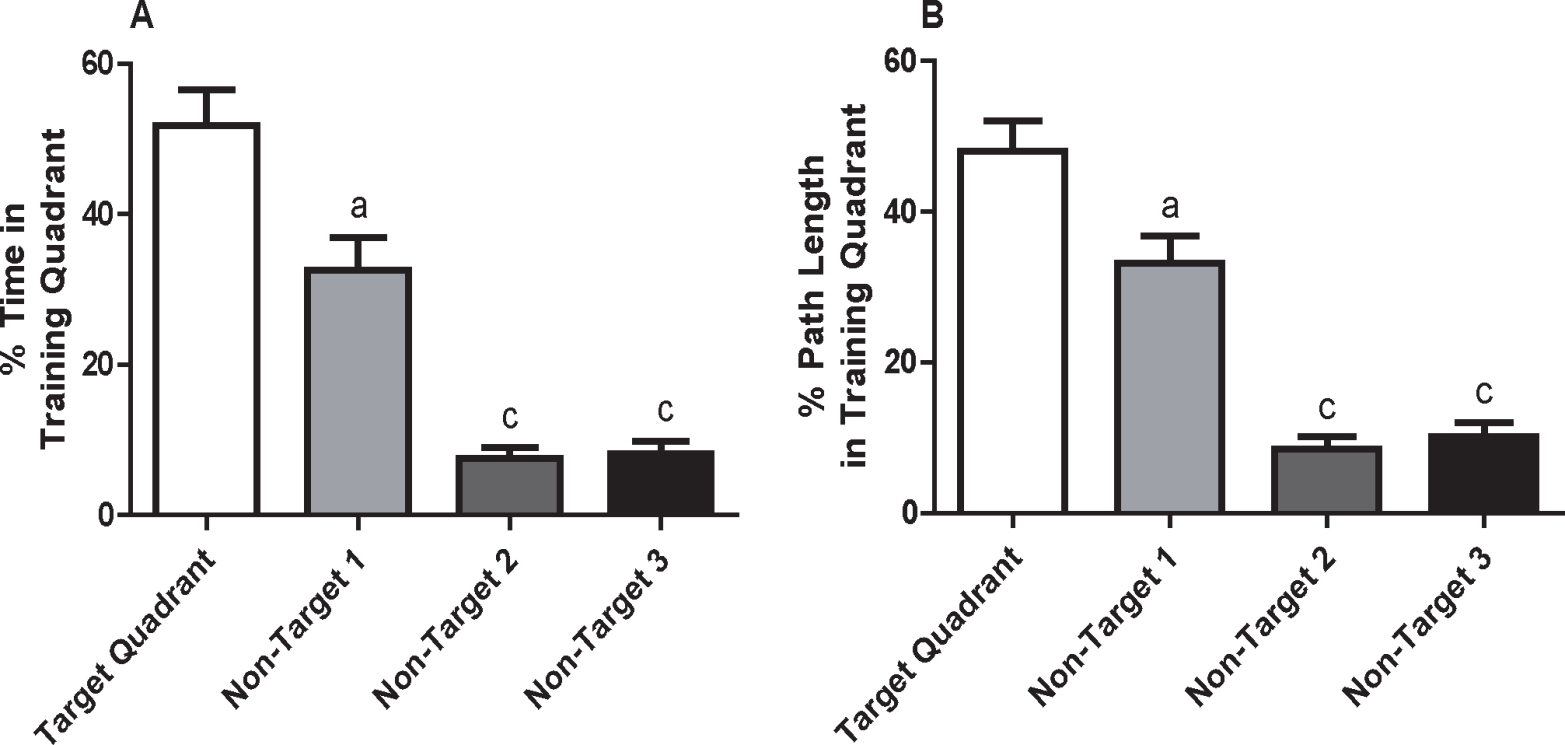
Weanling mice exhibit spatial memory after MWM training. Spatial memory was assessed in a probe trial administered on training day 8 with respect to: (A) percentage of time or (B) percentage of path length spent in the target quadrant relative to non-target quadrants. Data in panels A and B are presented as the mean ± SEM (n = 16 animals). Significantly different from target quadrant at ^a^ *p* < 0.05, ^c^ *p* < 0.001 as determined by repeated measures ANOVA with the Greenhouse-Geisser correction for LSD *post hoc* tests. Effect sizes: partial η^2^ for % time = 0.66; partial η^2^ for % path length = 0.68. Observed power: 100% for % time; 100% for % path length.

To assess spatial memory, a probe trial was administered on training day 8. Data were normally distributed. Repeated measures two-way ANOVA revealed no significant sex-dependent effects, so data from male and female weanlings were combined. After correcting for a violation of sphericity using the Greenhouse-Geisser correction, it was determined using a repeated measures one-way ANOVA that weanling mice spent a significantly increased percentage of time (*F*(1.5, 21.7) = 29.5, *p* < 0.0001) and path length (*F*(1.7, 25.8) = 32.1, *p* < 0.0001) in the target quadrant relative to the other non-target quadrants (Fig 2A and 2B, respectively). The effect sizes for both parameters were large (for time, partial η^2^ = 0.66; for % path length, partial η^2^ = 0.68). Observed power calculated by SPSS was 100% for both endpoints.

**Fig 3:**
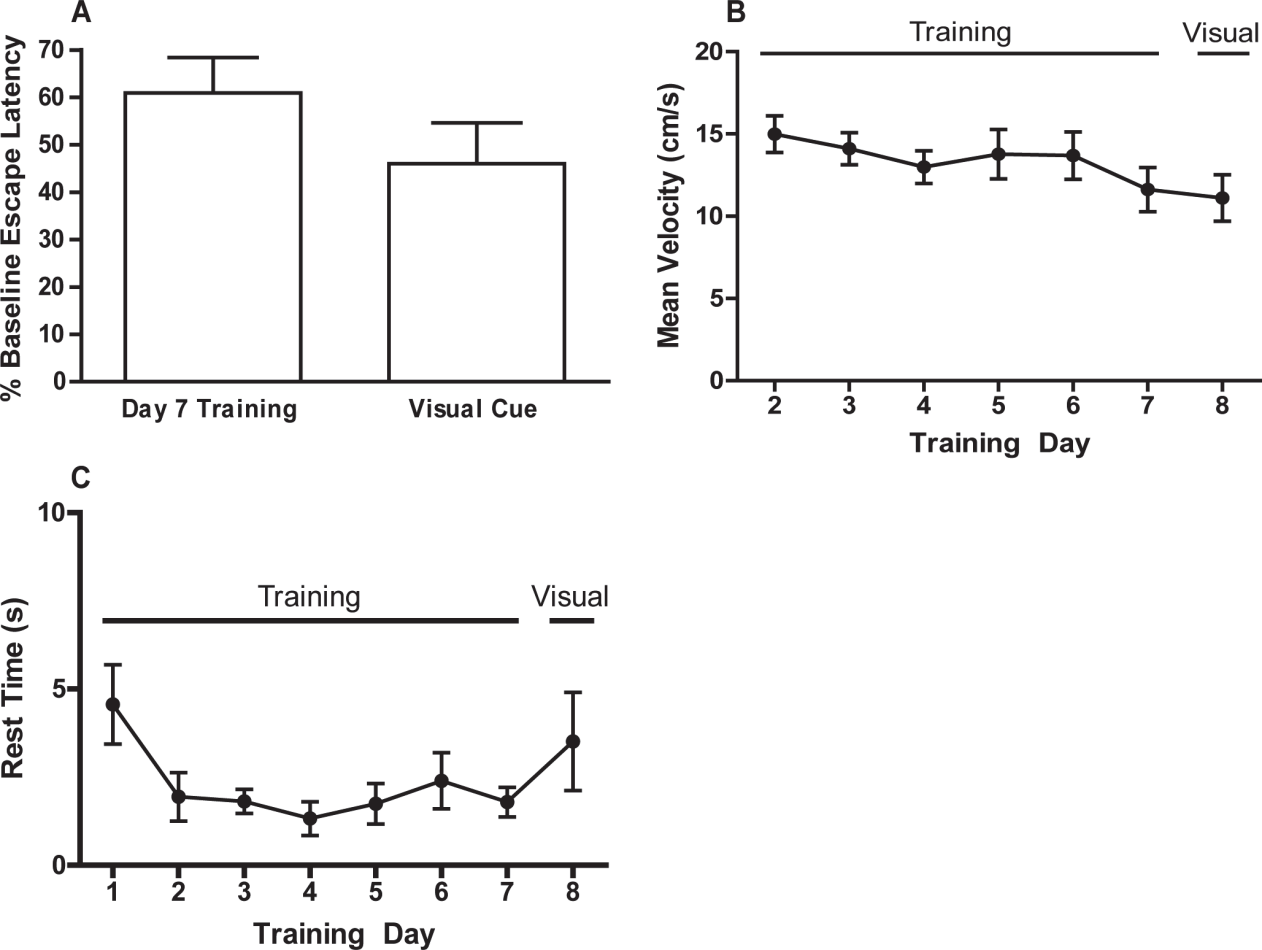
Results of the visual cue test. (A) Escape latency on d 7 of training and during the visual cue test expressed as a percentage of baseline escape latency (escape latency on the first training day). Additional parameters that influence performance in the MWM were assessed during the visual cue test including: (B) mean swim velocity and (C) rest time, both of which are presented as a function of training day. No statistically significant differences were identified using paired t-test (A) or repeated measures ANOVA (B,C).

Performance in the MWM is influenced by sensorimotor function and motivation, and these parameters can be assessed using a visual cue test [16]. The visual cue test was administered to weanling mice immediately following the probe test on day 8 of training. Data were normally distributed. T tests and repeated measures two-way ANOVA revealed no significant sex-dependent effects on visual cue parameters, so data from both sexes were combined. Escape latency during the visual cue test was decreased to 46% of the escape latency on the first day of training, which is referred to as the baseline escape latency (Fig 3A). This was a shorter escape latency than observed on d 7 of training, although the difference between escape latency during the visual cue test and training d 7 was not statistically significant by t test (*t*(28) = 1.31). Mean swim velocity did not change significantly over the course of training and was not significantly different in the visual cue test versus during training (*F*(6, 90) = 1.79) (Fig 3B). Weanlings spent an average of almost 5 s floating (rest time) during the first day of training. Rest time was reduced after the first day (Fig 3C). However, after correcting for violation of sphericity using the Greenhouse-Geisser correction, this difference was not significant as determined by repeated measures ANOVA (*F*(2.8, 42.8) = 2.59, ns). Several interesting behavioral phenomena were also observed during the visual cue test, including deflection off of the target (3 out of 16 animals), excessive floating or rest time (3 out of 16 animals), and failure to leave the initial quadrant (2 out of 16 animals).

### MWM training increases transcript levels of genes associated with synaptic plasticity

The mRNA levels of five genes associated with synaptic plasticity were measured by qPCR. Spinophilin (Spn) is a regulatory subunit for protein phosphatase 1, an enzyme associated with fine-tuning of synaptic strength. Spn is associated with spine density and morphogenesis, and is upregulated with environmental enrichment [30,31]. Activity-regulated cytoskeleton-associated protein (ARC) is an immediate early gene whose expression is modulated by activity. ARC plays a critical role in AMPA receptor trafficking and synaptic plasticity in general [32]. Neurogranin (RC3) is a critical determinant of the availability and localization of calmodulin. It is important for induction of long-term potentiation and cognitive function [33]. Homer1a and Homer1b/c are activity-inducible and constitutively expressed proteins that uncouple and couple intracellular and plasma membrane channels and fine-tune signaling between the two. Homer1 family proteins are involved in homeostatic and activity-dependent plasticity, spinogenesis, and synaptic plasticity [34,35]. Expression of these 5 genes was assessed at the mRNA level in the hippocampus, cortex and cerebellum because these 3 brain regions are the primary neuroanatomical substrates for learning in the Morris task [36–41].

**Fig 4:**
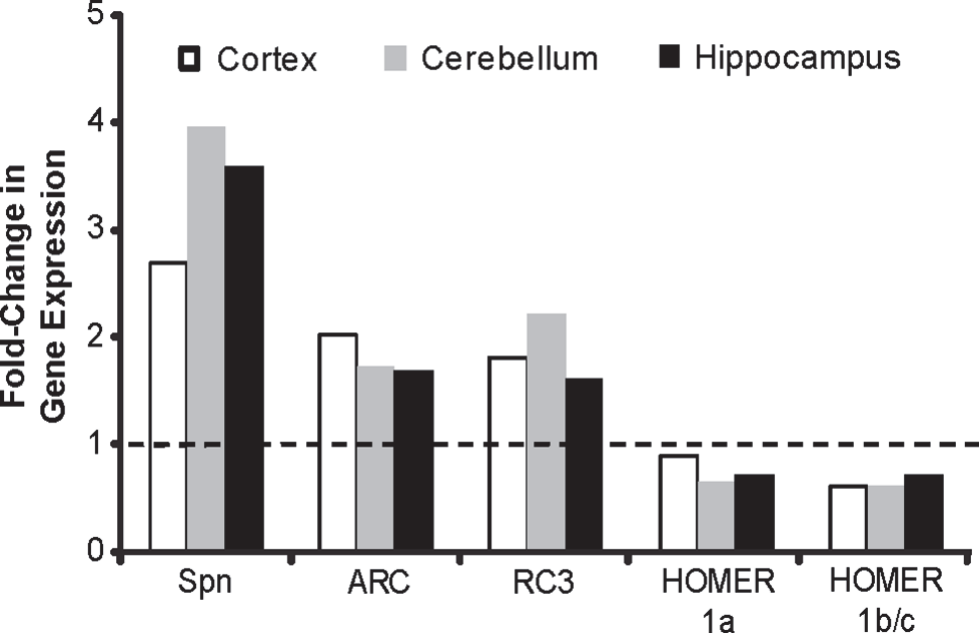
MWM training increases transcription of genes associated with synaptic plasticity in multiple brain regions. Transcript levels of spinophilin (Spn), activity-regulated cytoskeleton-associated protein (ARC), neurogranin (RC3), Homer1a and Homer1b/c were analyzed in total RNA harvested from the cortex, cerebellum, and hippocampus of weanling mice after behavioral studies were completed. Data are presented as fold-change in transcript expression relative to non-maze-trained littermates as calculated by the Pfaffl equation, normalized to the housekeeping gene 18S rRNA (n = 9–12 animals per group). The dashed line represents a fold-change of 1, which indicates no difference in gene expression between MWM-trained animals and untrained littermate controls.

Calculation of the fold-change in Spn, ARC, and RC3 mRNA using the Pfaffl equation indicated that all three transcripts were significantly increased in the cortex, cerebellum, and hippocampus of MWM-trained animals compared to untrained littermates (Fig 4). Further analyses using the REST2009 software similarly determined that MWM training significantly increased the expression of Spn, ARC and RC3 mRNA (Table 2). In contrast, MWM training did not significantly change transcript levels of Homer1a or Homer1b/c in any of the three brain regions investigated (Fig 4 and Table 2).

**Table 2:** REST2009 analysis of the effect of MWM training on transcript levels of plasticity-associated genes across different brain regions.

Gene	Cortex			Cerebellum			Hippocampus		
	Fold-Change ^a^	95% CI ^b^	p Value ^b^	Fold-Change	95% CI	p Value	Fold-Change	95% CI	p Value
Spn	3.53	0.84–38.03	**0.0001**	3.328	0.25–17.72	**0.001**	4.107	0.881–17.8	**0.0001**
ARC	2.099	0.75–6.41	**0.0001**	1.7	0.65–5.45	**0.004**	1.688	0.68–5.78	**0.006**
RC3	1.882	0.74–4.76	**0.001**	3.812	0.09–117.9	**0.032**	1.612	0.43–5.88	**0.040**
Homer1a	0.933	0.34–2.57	0.708	0.655	0.12–5.57	0.176	0.714	0.11–3.52	0.231
Homer1b/c	0.872	0.19–24.45	0.753	0.618	0.13–3.19	0.078	0.716	0.15–2.42	0.122

^a^Fold-change refers to the difference in expression between the target gene in MWM-trained weanling relative to untrained littermate controls (n = 8–12 animals).

^b^95% confidence intervals (CI) and p values were calculated by REST2009 software [28].

## Discussion

The major findings of this study include: (1) weanling mice exhibit spatial learning and memory in the MWM task; (2) there are no apparent sex differences in the performance of weanling mice in the MWM; and (3) MWM training upregulates transcription of genes associated with synaptic plasticity. The most direct evidence in support of the first conclusion is that training caused a significant decrease in escape latency and significant increase in both the percentage of swim time and path length spent in the target quadrant by training d 7 relative to performance on d 2 of training. Collectively, these data are interpreted as spatial learning because the mice require less time to find the platform and spend more time and path length in the target quadrant, indicating they have learned the platform location relative to the extra-maze visual cues [42]. The observed escape latencies are comparable to those recently reported in a study that demonstrated a reduction in MWM escape latency over a 5 d training period in PND 35 male offspring of exercised male mice [43]. Interestingly, the same study showed a much more modest decrease in escape latency of male offspring of unexercised male mice, indicating that performance of the weanling mouse in the MWM can be variable, and that the training paradigm presented here induces more robust spatial learning. It should also be noted that the escape latencies reported for weanling mice in the MWM do not decrease as rapidly or as robustly as has been reported for weanling rats [23,25,44] or adult mice [45–48] trained in the MWM.

An interesting observation from these initial experiments was that the percentage of time and path length spent in the target quadrant decreased on d 7 of training relative to values recorded for these same parameters on d 6 of training. A review of the original recordings provided several possible explanations for this unexpected reversal in performance between days 6 and 7 of training. On d 7, many weanlings exhibited increased thigmotaxis, whereas other weanlings located the platform very rapidly, but only after a period of floating, which translated to a decreased percentage of time and path length in the target quadrant on training d 7 relative to training d 6. As a result of these confounding behaviors, it is recommended that MWM studies with weanling mice be limited to a total of 6 training days.

Data collected during the probe trial revealed that weanling mice spent a significantly increased percentage of time and path length in the target quadrant compared to any other quadrant. Similar observations have been recently reported in two other studies of young mice trained in the Morris water maze starting at PND 35 [26,43], although the increase in time spent in the target quadrant during the probe trial documented in these earlier studies of slightly older mice was not as robust as we report here for weanlings. The percent increase in the time spent in the target quadrant during the probe trial reported for weanling rats [25,49,50] and adult mice [16,46,48] is of the same order of magnitude as we observed in weanling mice. This is interpreted as evidence of spatial memory since the weanlings spent significantly more of their total swimming time in proximity to the former platform location [42]. These data collectively suggest that weanling mice demonstrate spatial memory after training in the MWM.

The visual cue test revealed no significant sensorimotor deficiencies or motivational deficits in weanling mice. The percentage of baseline escape latency was lowest during the visual cue test, demonstrating an expected decrease in escape latency with the presentation of a visually conspicuous platform. Average visual cue escape latency was 29 s. This is higher than values reported for adult mice [48,51], which may be the result of confounding behaviors such as deflection of weanling mice off the platform and floating. Consistent with decreased escape latency, weanling mice exhibited a significantly lower cumulative path length in the visual cue test compared to the final day of training, indicating that the weanlings readily located the platform and swam directly to it. No significant differences were detected in mean swim velocity throughout training or in the visual cue test, indicating no overt issues in swimming ability. Mean swim velocity was between 10–20 cm/s in weanling mice in this study, which is similar to reported swim velocity values for PND 35 mice [52,53], weanling rats [25] and adult mice [45,46,51].

Several interesting behavioral phenomena were observed during the visual cue test. A majority of weanlings were able to locate the visible platform relatively quickly (escape latency ≤ 22 s; however there were a number of “slower” weanlings that required more time to mount the platform (escape latency ≥ 50 s). Several of these seemingly slower animals had located the platform, but deflected off of it rather than mounting it, despite being placed on the platform for 20 s immediately prior to the visual cue test. Determination of the latency to first contact with the platform was suggested that deflection increased the apparent escape latency in at least three weanlings. Deflection off the platform has been previously reported in adult mice trained in the MWM [54], and could possibly be due to intimidation by the novel visual stimulus Rest time averaged less than 5 s during every day of training, suggesting that the weanlings learned that floating would not expedite escape, and that weanlings had developed an association between locating and mounting the platform and escape from the pool. However, several of the weanlings with higher escape latencies during the visual cue test had a larger rest time than weanlings with lower escape latencies during the visual cue test. The weanlings with the highest escape latencies coincidentally had the largest rest times in the cohort. Only one weanling failed to enter the target quadrant during the visual cue test, but this weanling also never left the quadrant in which it was released. There is precedence in the literature for non-performing mice in the MWM task [54,55]. Overall, however, the results of the visual cue test confirm that weanling mice are capable of performing in the MWM since the same skills needed to perform in the visual cue test (adequate eyesight, motor skills needed to not only swim but also mount the platform, motivation to escape the water and association of the platform with escape) are required for performance in the training and probe trials of the MWM task [54].

Another interesting finding in our studies was the lack of significant sex-dependent differences in any behavioral endpoint assessed in the training trials, probe test, or visual cue test. A number of studies have demonstrated that adult male rodents acquire spatial learning tasks more efficiently than their age-matched female counterparts [15,56]. It is possible that our study was not sufficiently powered to detect sex differences; however, a lack of difference in MWM performance between male and female animals has been previously documented in weanling rats [25] and in rats at 6 months of age [57]. Puberty in mice generally begins after 4 weeks [58]. Training was initiated at P24, thus weanlings either had not entered puberty or sex hormones were too low to significantly impact behavior during the training period. Collectively, our findings and previous studies in weanling rats[25] suggest that age plays a role in sex-dependent differences in MWM performance.

Collectively, the data we obtained from the MWM task establish that weanling mice learn this navigational task and develop a spatial map that allows them to remember the platform location. It is believed that the molecular and cellular substrates of learning and memory include altered gene expression [59]. Thus, as a further readout of spatial learning and memory in weanling mice, we examined whether MWM training altered the expression of genes associated with synaptic plasticity. MWM increased transcription of Spn, ARC and RC3 as evidenced by significant increases in mRNA levels for these genes in the cortex, cerebellum, and hippocampus of MWM-trained weanlings compared to non-maze-trained control animals. The Pfaffl equation was used to calculate fold-changes in mRNA expression between maze-trained weanling mice and age- and sex-matched untrained littermates, and the significance of these fold-changes was confirmed using the REST2009 software. There is a high degree of variability in several of the samples as indicated by the broad spread of the upper and lower confidence interval limits, which is consistent with the variability observed in the raw Ct values, and also the variability in performance in the MWM. In contrast, MWM training did not significantly change expression of Homer1a or Homer1b/c in any of the three brain regions examined. The observation that Homer1a expression was not altered by MWM training may be explained by the fact that activity-dependent expression of this gene is transient. Vazdarjanova et al. measured a robust increase in Homer1a-positive cells in the hippocampus and cortex of rats, but only within minutes after environmental exploration [60]. Xiao et al. also measured upregulation of Homer1a mRNA after seizure; however, this was measured 3 h after stimulation [61]. Thus, we likely missed potential training-induced changes in Homer 1a transcription because our samples were collected 24 h after the last day of training in a 7 d training period. Consistent with our data, Xiao et al. did not observe any changes in Homer1b/c mRNA levels at 24 h after stimulation with the maximum electroconvulsive seizure model of activity [61]. No significant differences in mRNA expression between males and females were detected (results not shown), consistent with the lack of sex differences in MWM behavior.

Transcript levels in MWM-trained animals were compared to those in age- and sex-matched untrained littermates. While untrained littermates were handled identically to their trained counterparts with the exception of MWM training, it is possible that the upregulated expression of ARC, Spn and RC3 observed in the brains of the MWM trained animals are due to stress or physical exercise. Follow-up studies in our laboratory using mice of the same age and strain indicate that plasma levels of cortisol, a robust biomarker of stress, are not increased following MWM training using the same protocol described in this study (Barnhart and Lein, unpublished observations). These data suggest that stress is not a major factor driving the changes in transcript levels of ARC, Spn and RC3. Exercise has been shown to positively influence synaptic plasticity [62], but is not necessarily implicated in improvements in spatial learning paradigms [63]. While our data do not preclude a role for physical activity in the increased expression of synaptic plasticity genes in MWM-trained animals, the observation that Homer 1a and Homer 1 b/c were not upregulated 24 h following the last training session suggests that upregulation of ARC, Spn, and RC3 was relatively specific and not part of a general increase in gene expression due to a systemic influence such as physical activity. Changes in ARC, Spn, and RC3 expression have also been associated with performance in the MWM [64–66]. Thus, it is likely that increased levels of Spn, ARC, and RC3 mRNA observed in the brains of animals trained in the MWM provide molecular evidence of experience-dependent synaptic plasticity.

## Conclusions

In conclusion, these data are among the first to describe the use of the Morris water maze to investigate spatial learning and memory in the weanling mouse. Weanling mice exhibited significantly increased time and path length in the target quadrant and significantly decreased escape latency with MWM training, indicating that they are capable of maze learning as early as PND 24. Weanlings were also able to form spatial memories as indicated by significantly increased time and path length spent in the target quadrant and significantly decreased mean distance to the platform location during the probe trial. MWM training significantly increased the mRNA levels of genes associated with experience-dependent plasticity. In summary, our findings demonstrate that the Morris water maze can be used to assess spatial learning and memory in weanling mice, providing a potentially powerful experimental approach for examining the influence of genes, environmental factors and their interactions on the development of learning and memory.

## References

[1] Bloom B, Cohen RA, Freeman G. Summary health statistics for U.S. children: National Health Interview Survey, 2009. Vital Health Stat 10. 2010: 1–82. 21563639

[2] Landrigan PJ, Lambertini L, Birnbaum LS. A research strategy to discover the environmental causes of autism and neurodevelopmental disabilities. Environ Health Perspect. 2012; 120: a258–260. 10.1289/ehp.1104285 22543002 PMC3404655

[3] Grandjean P, Landrigan PJ. Neurobehavioural effects of developmental toxicity. Lancet Neurol. 2014; 13: 330–338. 10.1016/S1474-4422(13)70278-3 24556010 PMC4418502

[4] Trasande L, Liu Y. Reducing the staggering costs of environmental disease in children, estimated at $76.6 billion in 2008. Health Aff (Millwood). 2011; 30: 863–870. 10.1377/hlthaff.2010.1239 21543421

[5] Bellinger DC. A strategy for comparing the contributions of environmental chemicals and other risk factors to neurodevelopment of children. Environ Health Perspect. 2012; 120: 501–507. 10.1289/ehp.1104170 22182676 PMC3339460

[6] Grandjean P, Pichery C, Bellanger M, Budtz-Jorgensen E. Calculation of mercury's effects on neurodevelopment. Environ Health Perspect. 2012; 120: A452; author reply A452. 10.1289/ehp.1206046 23211440 PMC3548290

[7] Ricceri L, De Filippis B, Laviola G. Mouse models of Rett syndrome: from behavioural phenotyping to preclinical evaluation of new therapeutic approaches. Behav Pharmacol. 2008; 19: 501–517. 10.1097/FBP.0b013e32830c3645 18690105

[8] Kvajo M, McKellar H, Gogos JA. Avoiding mouse traps in schizophrenia genetics: lessons and promises from current and emerging mouse models. Neuroscience. 2012; 211: 136–164. 10.1016/j.neuroscience.2011.07.051 21821099 PMC3351555

[9] Yang XW, Lu XH. Molecular and cellular basis of obsessive-compulsive disorder-like behaviors: emerging view from mouse models. Curr Opin Neurol. 2011; 24: 114–118. 10.1097/WCO.0b013e32834451fb 21386675

[10] Mines MA, Yuskaitis CJ, King MK, Beurel E, Jope RS. GSK3 influences social preference and anxiety-related behaviors during social interaction in a mouse model of fragile X syndrome and autism. PLoS One. 2010; 5: e9706 10.1371/journal.pone.0009706 20300527 PMC2838793

[11] Crawley JN. Behavioral phenotyping strategies for mutant mice. Neuron. 2008; 57: 809–818. 10.1016/j.neuron.2008.03.001 18367082

[12] Li W, Dowd SE, Scurlock B, Acosta-Martinez V, Lyte M. Memory and learning behavior in mice is temporally associated with diet-induced alterations in gut bacteria. Physiol Behav. 2009; 96: 557–567. 10.1016/j.physbeh.2008.12.004 19135464

[13] Na ES, Nelson ED, Adachi M, Autry AE, Mahgoub MA, Kavalali ET, et al A mouse model for MeCP2 duplication syndrome: MeCP2 overexpression impairs learning and memory and synaptic transmission. J Neurosci. 2012; 32: 3109–3117. 10.1523/JNEUROSCI.6000-11.2012 22378884 PMC3835557

[14] Jiang YH, Pan Y, Zhu L, Landa L, Yoo J, Spencer C, et al Altered ultrasonic vocalization and impaired learning and memory in Angelman syndrome mouse model with a large maternal deletion from Ube3a to Gabrb3. PLoS One. 2010; 5: e12278 10.1371/journal.pone.0012278 20808828 PMC2924885

[15] D'Hooge R, De Deyn PP. Applications of the Morris water maze in the study of learning and memory. Brain Res Brain Res Rev. 2001; 36: 60–90. 11516773 10.1016/s0165-0173(01)00067-4

[16] Vorhees CV, Williams MT. Morris water maze: procedures for assessing spatial and related forms of learning and memory. Nat Protoc. 2006; 1: 848–858. 17406317 10.1038/nprot.2006.116 PMC2895266

[17] Barone S Jr., Das KP, Lassiter TL, White LD. Vulnerable processes of nervous system development: a review of markers and methods. Neurotoxicology. 2000; 21: 15–36. 10794382

[18] Ehman KD, Moser VC. Evaluation of cognitive function in weanling rats: a review of methods suitable for chemical screening. Neurotoxicol Teratol. 2006; 28: 144–161. 16414243 10.1016/j.ntt.2005.12.002

[19] Provost TL, Juarez de Ku LM, Zender C, Meserve LA. Dose- and age-dependent alterations in choline acetyltransferase (ChAT) activity, learning and memory, and thyroid hormones in 15- and 30-day old rats exposed to 1.25 or 12.5 PPM polychlorinated biphenyl (PCB) beginning at conception. Prog Neuropsychopharmacol Biol Psychiatry. 1999; 23: 915–928. 10509384 10.1016/s0278-5846(99)00035-4

[20] Corey DA, Juarez de Ku LM, Bingman VP, Meserve LA. Effects of exposure to polychlorinated biphenyl (PCB) from conception on growth, and development of endocrine, neurochemical, and cognitive measures in 60 day old rats. Growth Dev Aging. 1996; 60: 131–143. 9007564

[21] Rudy JW, Stadler-Morris S, Albert P. Ontogeny of spatial navigation behaviors in the rat: dissociation of "proximal"- and "distal"-cue-based behaviors. Behav Neurosci. 1987; 101: 62–73. 3828056 10.1037//0735-7044.101.1.62

[22] Goodlett CR, Peterson SD. Sex differences in vulnerability to developmental spatial learning deficits induced by limited binge alcohol exposure in neonatal rats. Neurobiol Learn Mem. 1995; 64: 265–275. 8564380 10.1006/nlme.1995.0009

[23] Jett DA, Kuhlmann AC, Farmer SJ, Guilarte TR. Age-dependent effects of developmental lead exposure on performance in the Morris water maze. Pharmacol Biochem Behav. 1997; 57: 271–279. 9164582 10.1016/s0091-3057(96)00350-4

[24] Jett DA, Navoa RV, Beckles RA, McLemore GL. Cognitive function and cholinergic neurochemistry in weanling rats exposed to chlorpyrifos. Toxicol Appl Pharmacol. 2001; 174: 89–98. 11446824 10.1006/taap.2001.9198

[25] Yang D, Kim KH, Phimister A, Bachstetter AD, Ward TR, Stackman RW, et al Developmental exposure to polychlorinated biphenyls interferes with experience-dependent dendritic plasticity and ryanodine receptor expression in weanling rats. Environ Health Perspect. 2009; 117: 426–435. 10.1289/ehp.11771 19337518 PMC2661913

[26] Salmaso N, Silbereis J, Komitova M, Mitchell P, Chapman K, Ment LR, et al Environmental enrichment increases the GFAP+ stem cell pool and reverses hypoxia-induced cognitive deficits in juvenile mice. J Neurosci. 2012; 32: 8930–8939. 10.1523/JNEUROSCI.1398-12.2012 22745493 PMC3399175

[27] Moser MB, Trommald M, Andersen P. An increase in dendritic spine density on hippocampal CA1 pyramidal cells following spatial learning in adult rats suggests the formation of new synapses. Proc Natl Acad Sci U S A. 1994; 91: 12673–12675. 7809099 10.1073/pnas.91.26.12673 PMC45501

[28] Pfaffl MW, Horgan GW, Dempfle L. Relative expression software tool (REST) for group-wise comparison and statistical analysis of relative expression results in real-time PCR. Nucleic Acids Res. 2002; 30: e36 11972351 10.1093/nar/30.9.e36 PMC113859

[29] Stamou M, Wu X, Kania-Korwel I, Lehmler HJ, Lein PJ. Cytochrome p450 mRNA expression in the rodent brain: species-, sex-, and region-dependent differences. Drug Metab Dispos. 2014; 42: 239–244. 10.1124/dmd.113.054239 24255117 PMC3912540

[30] Hu XL, Bergstrom SA, Brink M, Ronnback A, Dahlqvist P. Enriched environment increases spinophilin mRNA expression and spinophilin immunoreactive dendritic spines in hippocampus and cortex. Neurosci Lett. 2010; 476: 79–83. 10.1016/j.neulet.2010.04.007 20385205

[31] Feng J, Yan Z, Ferreira A, Tomizawa K, Liauw JA, Zhuo M, et al Spinophilin regulates the formation and function of dendritic spines. Proc Natl Acad Sci U S A. 2000; 97: 9287–9292. 10922077 10.1073/pnas.97.16.9287 PMC16860

[32] Shepherd JD, Bear MF. New views of Arc, a master regulator of synaptic plasticity. Nat Neurosci. 2011; 14: 279–284. 10.1038/nn.2708 21278731 PMC8040377

[33] Diez-Guerra FJ. Neurogranin, a link between calcium/calmodulin and protein kinase C signaling in synaptic plasticity. IUBMB Life. 2010; 62: 597–606. 10.1002/iub.357 20665622

[34] Gerstein H, O'Riordan K, Osting S, Schwarz M, Burger C. Rescue of synaptic plasticity and spatial learning deficits in the hippocampus of Homer1 knockout mice by recombinant Adeno-associated viral gene delivery of Homer1c. Neurobiol Learn Mem. 2012; 97: 17–29. 10.1016/j.nlm.2011.08.009 21945599 PMC3496399

[35] Sala C, Futai K, Yamamoto K, Worley PF, Hayashi Y, Sheng M. Inhibition of dendritic spine morphogenesis and synaptic transmission by activity-inducible protein Homer1a. J Neurosci. 2003; 23: 6327–6337. 12867517 10.1523/JNEUROSCI.23-15-06327.2003 PMC6740555

[36] Kolb B, Cioe J. Recovery from early cortical damage in rats, VIII. Earlier may be worse: behavioural dysfunction and abnormal cerebral morphogenesis following perinatal frontal cortical lesions in the rat. Neuropharmacology. 2000; 39: 756–764. 10699442 10.1016/s0028-3908(99)00260-9

[37] Leggio MG, Molinari M, Neri P, Graziano A, Mandolesi L, Petrosini L. Representation of actions in rats: the role of cerebellum in learning spatial performances by observation. Proc Natl Acad Sci U S A. 2000; 97: 2320–2325. 10681456 10.1073/pnas.040554297 PMC15799

[38] McNamara RK, Skelton RW. The neuropharmacological and neurochemical basis of place learning in the Morris water maze. Brain Res Brain Res Rev. 1993; 18: 33–49. 8467349 10.1016/0165-0173(93)90006-l

[39] Morris RG, Frey U. Hippocampal synaptic plasticity: role in spatial learning or the automatic recording of attended experience? Philos Trans R Soc Lond B Biol Sci. 1997; 352: 1489–1503. 9368938 10.1098/rstb.1997.0136 PMC1692060

[40] Ricceri L, Usiello A, Valanzano A, Calamandrei G, Frick K, Berger-Sweeney J. Neonatal 192 IgG-saporin lesions of basal forebrain cholinergic neurons selectively impair response to spatial novelty in adult rats. Behav Neurosci. 1999; 113: 1204–1215. 10636299 10.1037//0735-7044.113.6.1204

[41] Save E, Poucet B. Involvement of the hippocampus and associative parietal cortex in the use of proximal and distal landmarks for navigation. Behav Brain Res. 2000; 109: 195–206. 10762689 10.1016/s0166-4328(99)00173-4

[42] D'Hooge R, De Deyn PP. Applications of the Morris water maze in the study of learning and memory. Brain Research Reviews. 2001; 36: 60–90. 11516773 10.1016/s0165-0173(01)00067-4

[43] Yin MM, Wang W, Sun J, Liu S, Liu XL, Niu YM, et al Paternal treadmill exercise enhances spatial learning and memory related to hippocampus among male offspring. Behav Brain Res. 2013; 253: 297–304. 10.1016/j.bbr.2013.07.040 23916757

[44] Akers KG, Candelaria-Cook FT, Rice JP, Johnson TE, Hamilton DA. Cued platform training reveals early development of directional responding among preweanling rats in the Morris water task. Dev Psychobiol. 2011; 53: 1–12. 10.1002/dev.20480 20687138

[45] Varvel SA, Lichtman AH. Evaluation of CB1 receptor knockout mice in the Morris water maze. J Pharmacol Exp Ther. 2002; 301: 915–924. 12023519 10.1124/jpet.301.3.915

[46] Malleret G, Hen R, Guillou JL, Segu L, Buhot MC. 5-HT1B receptor knock-out mice exhibit increased exploratory activity and enhanced spatial memory performance in the Morris water maze. J Neurosci. 1999; 19: 6157–6168. 10407051 10.1523/JNEUROSCI.19-14-06157.1999 PMC6783095

[47] Florian C, Roullet P. Hippocampal CA3-region is crucial for acquisition and memory consolidation in Morris water maze task in mice. Behav Brain Res. 2004; 154: 365–374. 15313024 10.1016/j.bbr.2004.03.003

[48] Logue SF, Paylor R, Wehner JM. Hippocampal lesions cause learning deficits in inbred mice in the Morris water maze and conditioned-fear task. Behav Neurosci. 1997; 111: 104–113. 9109628 10.1037//0735-7044.111.1.104

[49] Jeltsch H, Bertrand F, Lazarus C, Cassel JC. Cognitive performances and locomotor activity following dentate granule cell damage in rats: role of lesion extent and type of memory tested. Neurobiol Learn Mem. 2001; 76: 81–105. 11525255 10.1006/nlme.2000.3986

[50] Rutten A, van Albada M, Silveira DC, Cha BH, Liu X, Hu YN, et al Memory impairment following status epilepticus in immature rats: time-course and environmental effects. Eur J Neurosci. 2002; 16: 501–513. 12193194 10.1046/j.1460-9568.2002.02103.x

[51] Frick KM, Stillner ET, Berger-Sweeney J. Mice are not little rats: species differences in a one-day water maze task. Neuroreport. 2000; 11: 3461–3465. 11095500 10.1097/00001756-200011090-00013

[52] Woods R, Vallero RO, Golub MS, Suarez JK, Ta TA, Yasui DH, et al Long-lived epigenetic interactions between perinatal PBDE exposure and Mecp2308 mutation. Hum Mol Genet. 2012; 21: 2399–2411. 10.1093/hmg/dds046 22343140 PMC3349420

[53] Ta TA, Koenig CM, Golub MS, Pessah IN, Qi L, Aronov PA, et al Bioaccumulation and behavioral effects of 2,2',4,4'-tetrabromodiphenyl ether (BDE-47) in perinatally exposed mice. Neurotoxicol Teratol. 2011; 33: 393–404. 10.1016/j.ntt.2011.02.003 21334437 PMC3543834

[54] Vorhees CV, Williams MT. Morris water maze: procedures for assessing spatial and related forms of learning and memory. Nature Protocols. 2006; 1: 848–858. 17406317 10.1038/nprot.2006.116 PMC2895266

[55] Lipp HP, Wolfer DP. Genetically modified mice and cognition. Current Opinion in Neurobiology. 1998; 8: 272–280. 9635213 10.1016/s0959-4388(98)80151-7

[56] Brandeis R, Brandys Y, Yehuda S. The use of the Morris Water Maze in the study of memory and learning. Int J Neurosci. 1989; 48: 29–69. 2684886 10.3109/00207458909002151

[57] Bucci DJ, Chiba AA, Gallagher M. Spatial learning in male and female Long-Evans rats. Behav Neurosci. 1995; 109: 180–183. 7734074 10.1037//0735-7044.109.1.180

[58] Green EL. Biology of the laboratory mouse. Biology of the laboratory mouse. 1966.

[59] Malenka RC. Synaptic plasticity: The brain's response to experience. Neuroscience Research. 2008; 61: S1–S1. 19391186

[60] Vazdarjanova A, McNaughton BL, Barnes CA, Worley PF, Guzowski JF. Experience-dependent coincident expression of the effector immediate-early genes arc and Homer 1a in hippocampal and neocortical neuronal networks. J Neurosci. 2002; 22: 10067–10071. 12451105 10.1523/JNEUROSCI.22-23-10067.2002 PMC6758761

[61] Xiao B, Tu JC, Petralia RS, Yuan JP, Doan A, Breder CD, et al Homer regulates the association of group 1 metabotropic glutamate receptors with multivalent complexes of homer-related, synaptic proteins. Neuron. 1998; 21: 707–716. 9808458 10.1016/s0896-6273(00)80588-7

[62] Wang Z, van Praag H (2012) Exercise and the brain: neurogenesis, synaptic plasticity, spine density, and angiogenesis Functional Neuroimaging in Exercise and Sport Sciences: Springer pp. 3–24.

[63] Rhodes JS, van Praag H, Jeffrey S, Girard I, Mitchell GS, Garland T Jr., et al Exercise increases hippocampal neurogenesis to high levels but does not improve spatial learning in mice bred for increased voluntary wheel running. Behav Neurosci. 2003; 117: 1006–1016. 14570550 10.1037/0735-7044.117.5.1006

[64] Huang FL, Huang KP, Wu J, Boucheron C. Environmental enrichment enhances neurogranin expression and hippocampal learning and memory but fails to rescue the impairments of neurogranin null mutant mice. J Neurosci. 2006; 26: 6230–6237. 16763030 10.1523/JNEUROSCI.1182-06.2006 PMC6675199

[65] Plath N, Ohana O, Dammermann B, Errington ML, Schmitz D, Gross C, et al Arc/Arg3.1 is essential for the consolidation of synaptic plasticity and memories. Neuron. 2006; 52: 437–444. 17088210 10.1016/j.neuron.2006.08.024

[66] Hongpaisan J, Alkon DL. A structural basis for enhancement of long-term associative memory in single dendritic spines regulated by PKC. Proc Natl Acad Sci U S A. 2007; 104: 19571–19576. 18073185 10.1073/pnas.0709311104 PMC2148330

